# The Tragedy of a Strong Muscle and a Weak Heart: Complications of Anabolic-Androgenic Steroid Misuse

**DOI:** 10.7759/cureus.17389

**Published:** 2021-08-23

**Authors:** Naga Vaishnavi Gadela, Hamza Coban, Evan Wasserman, Evan Schreyer, Abhishek Jaiswal

**Affiliations:** 1 Internal Medicine, University of Connecticut, Farmington, USA; 2 Neurology, University of Connecticut, Farmington, USA; 3 Cardiology, Hartford Hospital, Hartford, USA

**Keywords:** non-ischemic cardiomyopathy, anabolic-androgenic steroids, stroke, biventricular failure, cardiogenic shock

## Abstract

Misuse of anabolic-androgenic steroids (AAS) to boost performance and appearance is rising in the United States (US) with approximately one million people experiencing dependence. Long-term AAS misuse can lead to cardiovascular pathology but rarely cardiogenic shock. We report the case of an acute ischemic stroke secondary to an intracardiac thrombus in a patient with biventricular failure and cardiogenic shock associated with AAS misuse. Discontinuation of AAS and institution of guideline-directed medical therapy might reverse AAS-related heart failure.

## Introduction

The use of exogenous anabolic-androgenic steroids (AAS) is increasing among young athletes and has been implicated as a cause of various cardiovascular adverse effects such as myocardial infarction, dilated cardiomyopathy, and sudden cardiac death from malignant ventricular arrhythmias [1,2]. Although the literature regarding the potential for ventricular recovery following discontinuation of the offending agent is sparse, we report a case of a patient who presented with biventricular failure and cardiogenic shock associated with AAS misuse and demonstrated improvement in cardiac function following the discontinuation of drug and initiation of goal-directed medical therapy for heart failure.

## Case presentation

**Figure 1:**
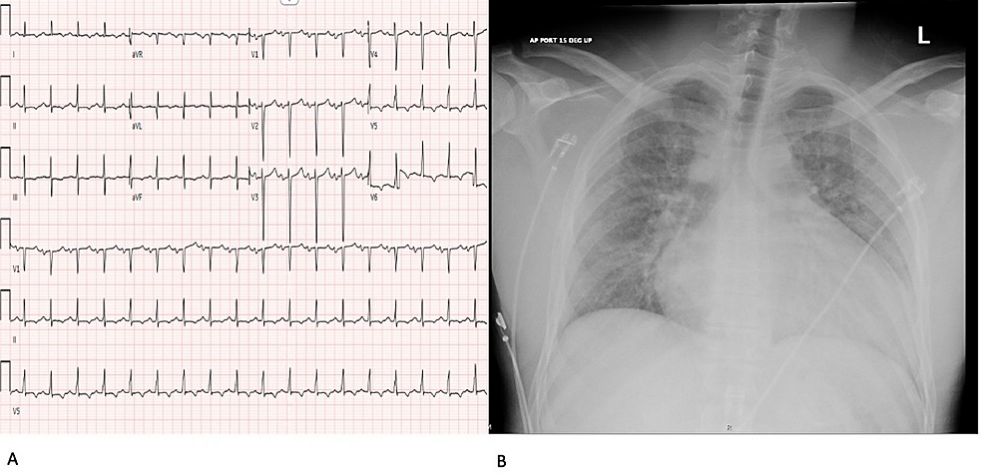
A: Initial EKG demonstrating sinus tachycardia; B: Chest radiograph showing interstitial edema and enlarged cardiac silhouette

A 41-year-old male with no significant past medical history presented with an acute onset speech difficulty and right-sided weakness. His initial assessment revealed a blood pressure of 140/80 mm Hg and a heart rate of 150 beats per minute. He was noted to have fluent, nonsensical speech with impaired comprehension, and right-sided weakness. The patient received intravenous tissue plasminogen activator after an emergency head CT showed no intracranial bleeding. Subsequently, he underwent endovascular thrombectomy with successful revascularization of the left middle cerebral artery. However, during this time, the patient developed progressive respiratory distress and hypotension. EKG revealed sinus tachycardia (Figure 1 A) and urgent chest radiography showed pulmonary edema (Figure 1 B). 

**Figure 2:**
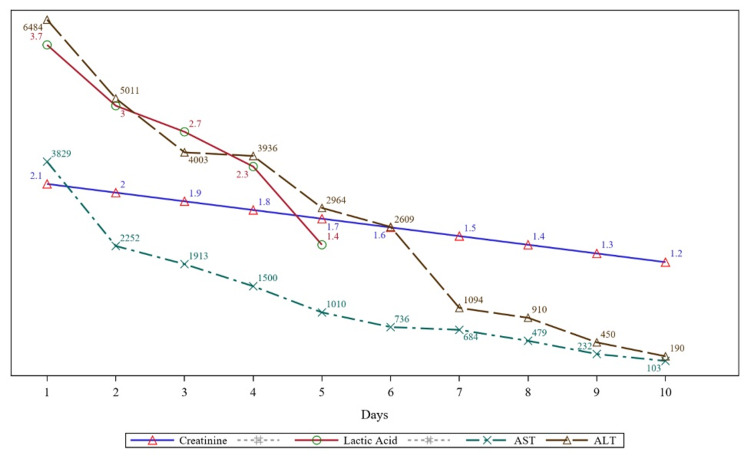
The trend of creatinine, lactic acid, alanine aminotransferase, and aspartate aminotransferase. ALT: Alanine aminotransferase, AST: Aspartate aminotransferase

An urgent bedside transthoracic EKG revealed a severely dilated left ventricle (LV) with an apical thrombus, global hypokinesis with an ejection fraction (EF) of 20%. Laboratory investigations showed a normal troponin level, serum lactic acid of 3.7 mmol/L, creatinine of 2.1mg/dl, aspartate aminotransferase of 3829 U/L, alanine aminotransferase of 5840 U/L, mildly elevated coagulation markers with prothrombin time of 15.7 seconds, and international normalized ratio (INR) of 1.4 (Figure 2).

During this time, the patient was found to have worsening right-sided neglect along with new right-homonymous hemianopia. A head CT scan showed an acute stroke in the posterior cerebral artery territory. The patient was started on heparin infusion due to recurrent thromboembolism. He was also initiated on dobutamine and furosemide infusion for his cardiogenic shock. 

After further discussions with the family, it was found that the patient had been using supplements including nandrolone, testosterone, and vitamin B12 for more than 10 years. Moreover, he was experiencing progressive dyspnea for a couple of weeks leading up to his hospitalization. Coronary angiography and cardiac MRI were proposed but the patient declined further workup. The patient continued to improve clinically and, subsequently, guideline-directed medical therapy with low dose metoprolol succinate, losartan, and spironolactone was initiated. Six months later, ejection fraction (EF) improved to 35% and the patient reported New York Heart Association (NYHA) class I-II functional capacity. The neurological function also improved with minimal motor deficits on the right upper extremity and right homonymous hemianopsia. 

## Discussion

**Figure 3:**
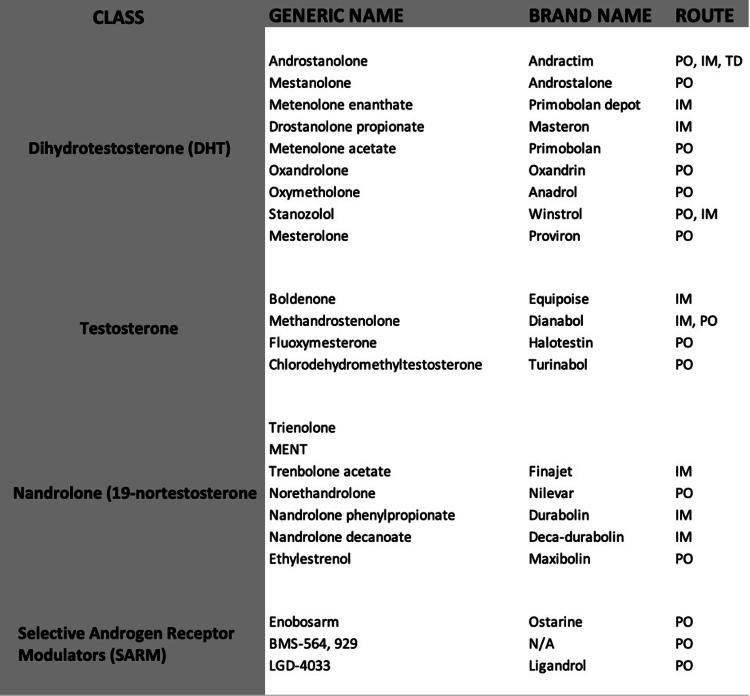
Commonly available anabolic-androgenic steroids IM: Intramuscular, PO: Per oral, TD: Transdermal

AAS are synthetic derivatives of testosterone, often used to manage hypogonadism and cachexia from various chronic conditions (Figure 3). Short-term AAS use can increase muscle strength, consequentially leading to long-term misuse [1]. It is likely that the actual untoward effects are underestimated as the relatively low doses administered in controlled settings do not approximate the higher doses used by illicit users. Moreover, the possibility of co-consumption of other agents and the fact that AAS misuse is not self-reported makes it challenging to study the full spectrum of effects.

Here, we present the first case of AAS-related cardiomyopathy presenting as an acute stroke in the setting of LV thrombus and cardiogenic shock. In our patient, although chronic resistance training may have caused structural cardiac alterations, functional impairment is infrequent with resistance training itself, and the AAS-associated cardiovascular effects may be causal.

**Figure 4:**
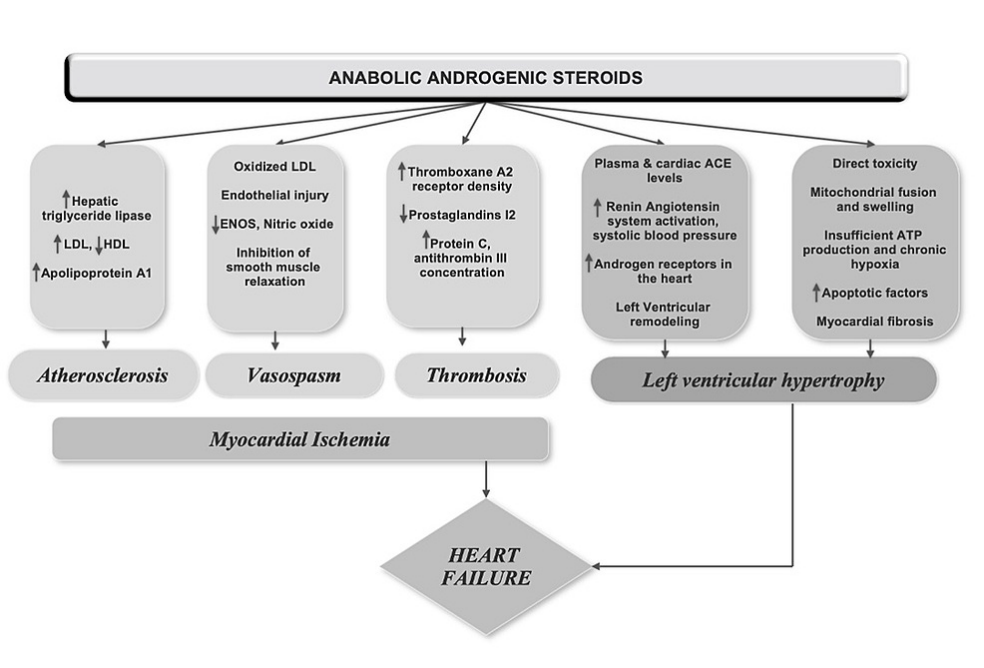
Pathophysiology of anabolic-androgenic steroid-induced heart failure LDL: Low-density lipoprotein, HDL: High-density lipoprotein, ENOS: Endothelial nitric oxide synthase, ACE: Angiotensin-converting enzyme, ATP: Adenosine triphosphate

The pathways delineating the effect of AAS are not well explored and several mechanisms have been proposed (Figure 4). Accelerated atherogenesis can be seen as early as one week after AAS use and increase the risk of coronary artery disease by three to six times [3]. Indeed, AAS users demonstrated a higher coronary plaque volume and the severity appeared to correlate with the duration of use [4]. Direct myocyte injury due to the release of apoptogenic factors causes disintegration of cardiomyocytes with changes similar to early heart failure [5]. This was corroborated by the myocardial autopsy of two bodybuilders [6]. A dose-dependent effect of AAS on LV hypertrophy (LVH) mediated by an androgen-receptor along with the activation of the renin-angiotensin system can contribute to cardiac remodeling with the presence of LVH, higher LV mass index, reduced LV systolic and diastolic function observed in long-term users. Besides, current users were found to have reduced LVEF and diastolic function compared with ex-users, suggesting reversibility [4,7-8]. While LV thrombus is not surprising in the presence of severely reduced LVEF, AAS itself promotes a hypercoagulable state by increasing the production of thromboxane A2 and decreasing prostaglandins [9].

Although confirmatory testing with serum/urine level of AAS has not been performed in our patient, his clinical presentation and corroboratory history from the family point towards AAS as the most likely etiology. Moreover, our patient had significant clinical improvement as well as improvement in his EF without revascularization, which points away from ischemic cardiomyopathy as a likely cause. Definitive management involves AAS cessation and guideline-directed medical therapy for heart failure. 

## Conclusions

Our case highlights the importance of a detailed evaluation of each patient’s clinical history to help identify (reversible) risk factors for heart failure and to provide appropriate treatment. Our patient demonstrated significant clinical improvement with discontinuation of AAS and institution of guideline-directed medical therapy. Long-term AAS misuse can lead to congestive heart failure and associated complications. As heart failure might reverse with discontinuation of AAS, prompt recognition, and reinforcement of abstinence from AAS should be pursued.
